# Enriched Environment Induces Sex-Specific Changes in the Adult Neurogenesis, Cytokine and miRNA Expression in Rat Hippocampus

**DOI:** 10.3390/biomedicines11051341

**Published:** 2023-05-02

**Authors:** Anna Vinogradova, Maria Sysova, Polina Smirnova, Maria Sidorova, Andrei Turkin, Ekaterina Kurilova, Oksana Tuchina

**Affiliations:** Educational and Scientific Cluster “Institute of Medicine and Life Sciences (MEDBIO)”, Immanuel Kant Baltic Federal University, 14 A. Nevskogo str., 236016 Kaliningrad, Russia

**Keywords:** enriched environment, neurogenesis, IL-10, miRNA, hippocampus, rats

## Abstract

An enriched environment stimulates adult hippocampal plasticity, but the exact cellular and molecular mechanisms are complex, and thus a matter of debate. We studied the behavior and hippocampal neurogenesis in adult male and female Wistar rats that were housed in an enriched environment (EE) for two months. Both EE males and females performed better than control animals in a Barnes maze, meaning that EE enhances spatial memory. However, the expression levels of neurogenesis markers KI67, DCX, Nestin, and Syn1 increased only in EE females, while in EE males only KI67 and BDNF were higher than in the corresponding control. The number of DCX+ neurons on brain slices increased in the dentate gyrus of EE females only, i.e., the level of adult hippocampal neurogenesis was increased in female but not in male rats. The level of anti-inflammatory IL-10 and signaling pathway components was upregulated in EE females. Of 84 miRNAs tested, in the hippocampi of EE female rats we detected upregulation in the expression levels of 12 miRNAs related to neuronal differentiation and morphogenesis, while in EE males four miRNAs were upregulated and involved in the regulation of cell proliferation/differentiation, and one was downregulated and associated with the stimulation of proliferation. Taken altogether, our results point to sex-specific differences in adult hippocampal plasticity, IL-10 expression, and miRNA profiles induced by an enriched environment.

## 1. Introduction

The environment we live in shapes the way we think and behave, but the exact cellular and molecular mechanisms that underlie these changes are complex and thus a matter of debate. In a series of studies in the early 1960s, M. R. Rosenzweig and coauthors demonstrated that increasing the environmental complexity in animal housing resulted in altered cholinesterase activity in rat cerebral cortex as well as significantly increased cortex weight [1,2]. These were the first experiments showing adult brain plasticity as a result of environmental enrichment. Ferchmin and Bennett (1975) later demonstrated that in order for these changes to appear, direct contact with an enriched environment is needed (i.e., personal experience), because rats that were housed under standard conditions but had the opportunity to observe animals in an enriched environment did not develop any of the environmentally-induced changes in their brains [3]. Normally the protocol of an enriched environment includes three main components that stimulate (1) physical activity (additional space and/or wheels for voluntary running), (2) exploratory activity (mazes, toys), and (3) social interactions (the possibility of contact with other mice/rats of one’s own and/or the opposite sex). Rosenzweig et al. (1978) noted that the combination of these three components is an essential feature of an enriched environment, since, for instance, social enrichment alone does not change the thickness of the cerebral cortex, acetylcholinesterase activity, or the total amount of RNA in the rat cortex [4]. Since then, the number of studies devoted to the role of an enriched environment has continuously increased, featuring environmentally-induced changes in the cortex, amygdala, hippocampus, and other brain regions. The hippocampus is critical for spatial memory encoding [5], which is believed to be essential in order to navigate a more complex environment, and it is also widely known as one of the neurogenic niches in the adult mammalian brain [6,7]. The development of new tools and techniques allowed us to see that environmental enrichment induces changes in the firing patterns of CA1 hippocampal neurons [8], enhances histone acetylation of glutamate receptors [9], stimulates cognitive function and BDNF signaling [10] and adult neurogenesis [11], as well as reduces inflammation in the rodent hippocampus caused by respiratory infection [12] or stroke [13] and, moreover, it restores the connectivity between the hippocampus and the medial prefrontal cortex, which was weakened by chronic stress [14]. However, being complex, an enriched environment does not have a strict definition even for rodents, and its protocols may vary between laboratories, decreasing the reproducibility of the results [15]. For example, some protocols include changing objects (usually, toys or feeding places) a few times a week that might affect the exploratory activity, or include contacts with animals of the opposite sex, not to mention that the size of the cages for environmental enrichment also vary. Another caveat is that an enriched environment might not improve all types of learning or increase measures of all brain structures, as it does in the cerebral cortex [16]. Although normally the enrichment of an environment has beneficial effects on many aspects of brain function, there are studies showing evidence of increased anxiety [17,18] and corticosterone levels [19] as a result of housing rodents in an enriched environment. An elevated level of anxiety is often associated with chronic stress and a decreased level of adult hippocampal neurogenesis [20]. Another interesting and often missed point is possible sexual differences in environment-induced brain plasticity. Recent studies emphasize the importance of including both sexes in studies of hippocampal plasticity [21], since the hippocampus is highly complex and modifiable by internal (such as sexual hormones) as well as external (environmental) inputs. We have studied sex-differences in behavior, hippocampal neurogenesis, cytokine expression, and miRNA profiles in rats housed in an enriched environment.

## 2. Materials and Methods

### 2.1. Animals and Enriched Environment Protocol

**Figure 1:**
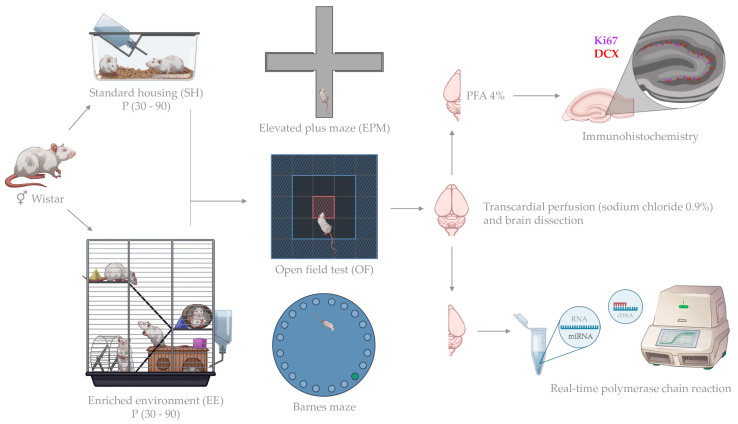
Experimental workflow.

Male and female Wistar rats were used in the study. All animals were housed in standard environmental conditions (23 ± 2 °C; 12 h/12 h dark/light cycle in polypropylene cages) with water and food provided ad libitum in the animal care facility at Immanuel Kant Baltic Federal University in Kaliningrad, Russia. All experiments including the number of animals used in the study were approved by the Independent Ethical Committee of the Clinical Research Center at IKBFU, Kaliningrad, protocol 27/2021. At the age of 1 month (P30), the animals were divided into groups: control (SH, standard housing in polypropylene cages 35 × 50 × 20 cm, 2 rats/cage) and an enriched environment (EE, animal housing in three-tiered cages from stainless steel wire, 46 × 76 × 78 cm, equipped with side wheels, ladders and toys, the latter were changed every three days, 6–9 rats/cage). The rats of the same sex were kept in EE or SH conditions for two months. At the age of three months (P90), animals from the control (SH) and experimental (EE) groups underwent behavioral tests (Supplementary Table S1). The experimental workflow is represented on Figure 1.

### 2.2. Behavior Phenotyping

Before the behavioral tests (at least 30 min in advance), the cages with rats were placed in the behavioral testing facility in order for the animals to get used to the new environment. At least 2 h passed between the different behavioral tests. All mazes were first wiped with a damp cloth and then with a 70% ethanol wipe, and the room was ventilated between tests for at least 5 min.

Open field test (OF). In order to test the exploratory behavior and general activity in the OF, rats were individually placed into the corner of an open arena (100 cm long × 100 cm wide × 50 cm high borders) under dim lighting conditions and allowed to move freely for 5 min with their activities videotaped using a GoPro HERO 9 Black camera. Time spent in the central area (20 × 20 cm) and on the periphery, animal activity (*n* = 23 females SH, *n* = 28 females EE, *n* = 24 males SH, *n* = 27 males EE) and latent period of exit to the center (*n* = 8 females SH, *n* = 10 females EE, *n* = 9 males SH, *n* = 6 males EE) were measured using the BehaviorCloud software (BehaviorCloud, San Diego, CA, USA). Some of the rats were tested before being placed in EE conditions, and their behavior was then compared with the results of the testing after spending 2 months in EE (*n* = 8 females SH, *n* = 6 females EE, *n* = 8 males SH, *n* = 7 males EE).

Elevated plus maze (EPM). The apparatus consisted of two opposing open arms (50 cm long × 10 cm wide × 1.5 cm high borders) and two opposing enclosed arms (50 cm long × 10 cm wide × 40 cm high borders) fixed to a central platform (10 × 10 cm) in a cross shape. The height of the maze was 100 cm above the floor. Each rat was placed onto the center area, heading toward an open arm, and videotaped for the following 5 min using the GoPro HERO 9 Black camera (*n* = 23 females SH, *n* = 23 females EE, *n* = 24 males SH, *n* = 23 males EE). Time spent in the open arms and enclosed arms of the maze as well as the number of entries to the sectors were analyzed using the RealTimer software (Open Science, Krasnogorsk, Russia).

Barnes maze. The Barnes maze consisted of a circular surface (122 cm in diameter) with up to 20 evenly spaced circular holes (10 cm in diameter) around its circumference. An escape box (30 × 15 × 12 cm) was positioned under one of the escape holes. Visual cues (colored triangle, square, circle and star) were placed around the maze in plain sight of the rat (20 cm from the maze). The activity of the rats was recorded with the GoPro HERO 9 Black camera, and the analysis of behavior was carried out using BehaviorCloud (BehaviorCloud, San Diego, CA, USA). We followed the Barnes maze procedure for spatial learning and memory analysis according to Pitts (2018) [22]. During the habituation and the acquisition phases, the escape time was considered, while during the probe phase, the time spent in the target sector was measured (*n* = 13 females SH, *n* = 16 females EE, *n* = 14 males SH, *n* = 17 males EE).

### 2.3. Brain Dissection

After conducting behavioral tests, we performed a transcardial perfusion. Before the perfusion, the animals were deeply anesthetized with isoflurane (Aesica Queenborough, Queenborough, UK). The use of the anesthetic was carried out in accordance with the safety instructions for working with volatile substances, therefore anesthesia was carried out in a desiccator in a fume hood. Decapitation was carried out after checking the effect of the anesthesia (characteristic signs: loss of consciousness, muscle relaxation, lack of pain sensitivity and slow, barely noticeable breathing). A 0.9% solution of sodium chloride was used to wash the cerebral vessels during perfusion. Brain isolation procedures were performed in a clean room using medical instruments and a blood disinfectant. After dissection, the brain was cut into hemispheres, and the hippocampus was isolated from one hemisphere for the subsequent polymerase chain reaction. The other hemisphere was fixed in 4% paraformaldehyde in a sodium phosphate buffer (PBS, pH 7.4) for at least 24 h, then placed in 30% sucrose and sectioned on a cryotome (KEDEE Instruments, Jinhua, China).

### 2.4. Real-Time Polymerase Chain Reaction

All reagents (if not stated otherwise) were obtained from Evrogen (Evrogen, Moscow, Russia). The hippocampus was crushed to a homogeneous state using a pestle homogenizer, then mixed with a solution of phenol and guanidine isothiocyanate Extract RNA. After 10 min of incubation at room temperature, 1/5 volume of chloroform was added to the solution, followed by centrifugation at a temperature of 4 °C and a speed of 12,000× *g*. The upper RNA-enriched aqueous phase was collected separately from the DNA and proteins in a new test tube. The resulting RNA solution was purified by alternate washing with absolute isopropyl and 80% ethyl alcohols. The RNA precipitate was then dissolved in highly purified water free of nucleases and nucleic acids. The RNA concentration was measured on a NanoPhotometer Pearl spectrophotometer (Implen, München, Germany). Total RNA in the amount of 1 μg was used to obtain complementary DNA using a reverse transcription kit according to the manufacturer’s instructions (Evrogen, Moscow, Russia). The resulting DNA was diluted with highly purified water for PCR to a final concentration of 10 ng/μL. The quantitative PCR was performed using gene-specific primers for KI67, DCX, Nestin, BDNF (*n* = 7 female SH, *n* = 5 female EE, *n* = 6 male SH, *n* = 6 male EE), IL-10 and IL-10RA (*n* = 6 female SH, *n* = 5 female EE, *n* = 6 male SH, *n* = 6 male EE), JAK1, STAT1, STAT3, STAT5A, and STAT5B (*n* = 5–7 female SH, *n* = 5 female EE, *n* = 6 male SH, *n* = 6 male EE), with GAPDH used as a reference gene. The sequence of primers used is shown in Table 1. The selection of primers, the analysis for self-complementarity, and the formation of hairpin structures were performed in the program PrimerQuest Tool and OligoAnalyzer https://www.idtdna.com/ (accessed on 17 February 2021). The mRNA sequences of the studied genes were obtained on the website of the National Center for Biotechnological Information https://www.ncbi.nlm.nih.gov/ (accessed on 17 February 2021). The complementarity of the selected primers to the mRNAs under study was verified in the Primer Blast program https://www.ncbi.nlm.nih.gov/tools/primer-blast/ (accessed on 17 February 2021). The synthesis of oligonucleotide primers was performed by Evrogen (Evrogen, Moscow, Russia). The formulation of the quantitative PCR reaction was performed on a CFX96 Real-Time System (BIO-RAD, Hercules, CA, USA). The data were analyzed using the ∆∆Ct method.

#### miRNA Extraction and Analysis

In order to analyze changes in the microRNA expression profiles of rats housed in EE and SH conditions, we prepared hippocampal tissue samples (*n* = 3 females SH, *n* = 3 females EE, *n* = 3 males SH, *n* = 3 males EE). MiRNAs were extracted from the tissue samples using the miRNeasy Micro Kit (Qiagen, Hilden, Germany) according to the supplier protocol. Briefly, 10 mg of sample/each rat was mixed with 700 µL of QIAzol Lysis reagent and incubated at room temperature for 5 min. The sample was then mixed with 140 µL of chloroform. After vortexing and incubating at room temperature for 5 min, we centrifuged the samples at 12,000× g at 4 °C for 15 min in order to separate them into three phases. The aqueous phase was then transferred into the new tube, and 1/5 volume of absolute ethanol was added. The RNeasy MinElute Spin Columns were used to wash and finally elute the RNA in 14 µL of RNase free water. No pooling of samples was performed. The first-strand complementary synthesis reaction (cDNA) was performed using the miScript II RT kit (Qiagen, Hilden, Germany) according to the supplier protocol. Briefly, 1 μg of isolated RNA was added to the cDNA master mix, which was composed of 5× miScript HiSpec Buffer, 10× miScript Nucleics Mix, miScript Reverse Transcriptase Mix, and water, to a total volume of 20 µL. The cDNA was incubated at 37 °C for 60 min, followed by 5 min incubation at 95 °C. For amplification we used rat cell differentiation and development miScript miRNA PCR arrays (MIRN-103Z, lot no. DX20.2-R1, Qiagen, Hilden, Germany) with a miScript SYBR Green PCR kit (Qiagen, Hilden, Germany) in a CFX96 system (BioRad, Hercules, CA, USA), following the cycling conditions recommended by the supplier protocol (15 min at 95 °C, followed by 40 cycles of 15 s at 94 °C, 30 s at 55 °C, and 30 s at 70 °C). GeneGlobe Data Analysis (Qiagen) was used for statistical analyses (https://geneglobe.qiagen.com/it/analyze, (accessed on 22 April 2022)). A global normalization was performed which included endogenous small RNA SNORD95. The significance was assessed by the 2–ΔΔCT method [24]. Changes were defined as ±1.5-fold up- and down-regulation compared with the control, standard/enriched environment conditions. An independent sample *t*-test (two-tail) was used to compare the data between groups. The significance was set at *p* ≤ 0.05. The expression of the following 84 microRNAs has been analyzed: rno-let-7a-5p, rno-let-7b-5p, rno-let-7c-5p, rno-let-7d-5p, rno-let-7e-5p, rno-let-7f-5p, rno-let-7i-5p, rno-miR-1-3p, rno-miR-100-5p, rno-miR-101a-3p, rno-miR-101a-5p, rno-miR-101b-3p, rno-miR-103-3p, rno-miR-106b-5p, rno-miR-10a-3p, rno-miR-10b-5p, rno-miR-122-5p, rno-miR-124-3p, rno-miR-125a-5p, rno-miR-125b-5p, rno-miR-126a-3p, rno-miR-130a-3p, rno-miR-132-3p, rno-miR-133b-3p, rno-miR-134-5p, rno-miR-137-3p, rno-miR-138-5p, rno-miR-140-5p, rno-miR-141-3p, rno-miR-142-3p, rno-miR-142-5p, rno-miR-144-3p, rno-miR-146a-5p, rno-miR-146b-5p, rno-miR-150-5p, rno-miR-15b-5p, rno-miR-16-5p, rno-miR-181a-5p, rno-miR-182, rno-miR-183-5p, rno-miR-185-5p, rno-miR-18a-5p, rno-miR-192-5p, rno-miR-194-5p, rno-miR-195-5p, rno-miR-196a-5p, rno-miR-203a-3p, rno-miR-205, rno-miR-206-3p, rno-miR-280a-3p, rno-miR-20a-5p, rno-miR-20b-3p, rno-miR-21-5p, rno-miR-210-3p, rno-miR-214-3p, rno-miR-215, rno-miR-218a-5p, rno-miR-219-5p, rno-miR-22-3p, rno-miR-222-3p, rno-miR-223-3p, rno-miR-23b-3p, rno-miR-24-3p, rno-miR-26a-5p, rno-miR-26b-5p, rno-miR-292-3p, rno-miR-310a-3p, rno-miR-320-3p, rno-miR-322-5p, rno-miR-33-5p, rno-miR-345-5p, rno-miR-375-3p, rno-miR-378a-3p, rno-miR-429, rno-miR-451-5p, rno-miR-488-3p, rno-miR-503-5p, rno-miR-541-3p, rno-miR-7a-5p, rno-miR-9a-5p, rno-miR-92a-3p, rno-miR-93-5p, rno-miR-96-5p, and rno-miR-99a-5p.

### 2.5. Immunohistochemistry

All reagents for immunohistochemistry were obtained from Paneco (Paneco, Moscow, Russia) if not stated otherwise. For immunohistochemistry, one hemisphere was fixed in fresh ice cold 4% paraformaldehyde solution for at least 24 h, then placed in 30% sucrose for at least 24 h, embedded in Tissue-Tek O.C.T. compound (Sakura Finetek, Torrance, CA, USA), and then sectioned on a cryotome (KEDEE Instruments, Zhejiang, China) in order to obtain 50 μm serial sections. Before staining, the sections were stored in PBS containing 0.01% sodium azide at 4 °C. The sections were then washed in PBS 3 times for 10 min, and then incubated in 5% fetal bovine serum (FBS) in PBS containing 0.3% Triton X-100 overnight. The sections were divided into strips and incubated with the following primary antibodies: anti-KI67 rabbit monoclonal (ThermoFisher Scientific, Waltham, MA, USA; MA514520, 1:500), anti-DCX rabbit polyclonal (ThermoFisher Scientific, Waltham, MA, USA; PA5-17428, 1:1000), and anti-NeuN mouse monoclonal (MerkMillipore, Burlington, MA, USA; MAB377, 1:1000) for 48h in 1% FBS on 0.3% PBST. After incubation with primary antibodies, sections were washed three times for 10 min in PBS and incubated with secondary antibodies: donkey anti-rabbit Alexa555 (Abcam, Cambridge, UK; ab150074, 1:1000), and donkey anti-mouse Alexa488 (Abcam, Cambridge, UK; ab150105, 1:1000) overnight. After the staining, all sections were washed three times in PBS for 10 min, counterstained with Hoechst 33,342 trihydrochloride (Molecular Probes, Eugene, OR, USA; 1542001, 1:1000), mounted on glass slides using Mowiol-based mounting medium, and examined under an Axio imager A2 fluorescent microscope (Carl Zeiss, Jena, Germany) and a LSM 780 confocal scanning microscope (Carl Zeiss, Jena, Germany) with ZEN 780 software. KI67+ and DCX+ cells were counted in the dorsal and ventral dentate gyrus in the subgranular zone under an Axio imager A2 fluorescent microscope (Carl Zeiss, Jena, Germany).

### 2.6. Statistical Analysis

The statistical analysis was performed using Prism 9 software (GraphPad, San Diego, CA, USA). The Shapiro–Wilk test was used in order to determine the normality of the variables. In the case of a normal distribution, a *t*-test was performed (in the case of two independent samples) or ANOVA (in the case of three or more independent samples) with the analysis of multiple comparisons by a Tukey’s test. The differences were considered statistically significant at *p* ≤ 0.05. In order to represent the data, bar charts were used, showing the sample mean ± standard error of the mean (SEM). The statistical significance is represented as follows: *p* ≤ 0.05 (*), *p* ≤ 0.01 (**), *p* ≤ 0.001 (***), *p* ≤ 0.0001 (****).

## 3. Results

### 3.1. EE Rats Showed a Decrease in Activity and Some Signs of Anxiety, but Performed Better Than SH Animals in the Barnes Maze

**Figure 2:**
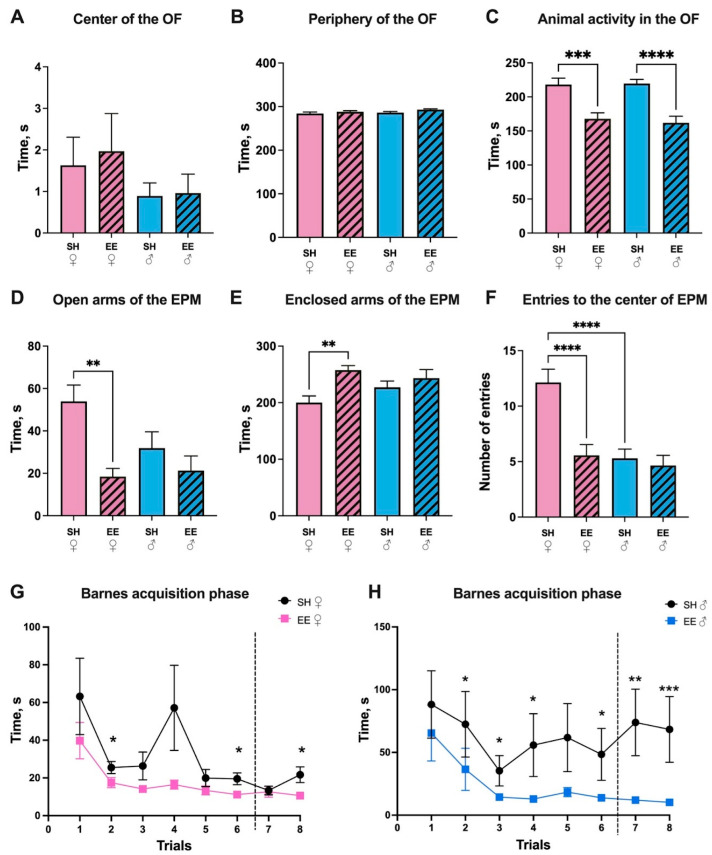
The effect of an enriched environment (EE) on the activity of female (pink) and male (blue) Wistar rats in the Open field (**A**–**C**), Elevated Plus maze (**D**–**F**) and Barnes maze (**G**,**H**) tests. (**A**) The time animals spent in the center of the OF, (**B**) in the periphery of the OF, (**C**) moving in the OF, (**D**) in the open arms of the EPM, (**E**) in the enclosed arms of the EPM, (**F**) the number of entries to the center of the EPM made by the animals, (**G**) the time female rats required in order to find shelter during acquisition trials (1–8) in the Barnes maze, (**H**) the time male rats required in order to find shelter during acquisition trials (1–8) in the Barnes maze. The dashed line represents an interval in training trials for 48 h. EE: enriched environment, SH: standard housing. ♂—males, ♀—females. A striped pattern of the bars refers to EE, solid fill—to SH conditions. The data is represented as means ± SEM; * *p* ≤ 0.05, ** *p* ≤ 0.01, *** *p* ≤ 0.001, **** *p* ≤ 0.0001 (ANOVA with a Tukey’s test for (**A**–**F**), and a Mann–Whitney test for SH/EE comparisons for each trial for (**G**,**H**).

Living in an enriched environment for two months did not influence rat behavior in the OF in terms of the time that the animals spent in the center (SH vs. EE females: *p* = 0.98; SH vs. EE males: *p* = 0.99) and in the periphery (SH vs. EE females: *p* = 0.69; SH vs. EE males: *p* = 0.26) of the maze (Figure 2A–C). Both males and females preferred to stay close to the borders of the maze, avoiding the center. Out of 23 SH females and 28 EE females, 8 and 10 animals entered the center of the maze, respectively (which is 34.8% and 35.7%, respectively), and out of 24 SH males and 27 EE males, nine and six animals entered the center, respectively (which is 37.5% and 22.2%). At the same time, the horizontal activity changed: EE rats spent significantly less time moving in the maze compared to the corresponding controls (SH vs. EE females: *p* = 0.0005; SH vs. EE males: *p* ≤ 0.0001). Thus, we can suggest that the exploratory behavior of EE rats decreased in terms of activity compared to the corresponding controls. At the same time, both the SH and EE rats preferred to avoid the center of the maze. In order to analyze anxiety-like behavior, we performed the EPM test (Figure 2D–F). Female EE rats spent significantly less time in the open arms of the EPM than SH females (*p* = 0.002), while for males there was no difference (*p* = 0.67); it is worth noting that there were no significant differences between SH females and males (*p* = 0.10). EE females spent more time in the enclosed arms of the EPM (*p* = 0.005), while there were no differences for males (*p* = 0.76). Interestingly, however, SH males and EE females made significantly fewer entries to the center of the maze compared to SH females (*p* ≤ 0.0001). Hence, in the EPM, both SH and EE rats preferred to stay in the enclosed arms of the maze, but only EE females showed significant differences compared to the corresponding control group. We found that the animals displayed signs of anxiety-like behavior. The K. Barnes maze test was conducted in three stages: the habituation phase, the acquisition phase (consisted of 8 trials), and the probe phase. During the habituation phase, the escape time did not differ in the EE and SH animals (*p* = 0.98 for females, and *p* = 0.71 for males). However, during the acquisition phase, EE females found the escape box significantly faster than SH females at two, six, and eight training sessions (*p* = 0.03, *p* = 0.03, *p* = 0.02, respectively), while EE males found the escape box faster than SH males during two, three, four, six, seven, and eight training sessions (*p* = 0.02, *p* = 0.04, *p* = 0.02, *p* = 0.047, *p* = 0.001, *p* = 0.003, respectively) (Figure 2G,H). The time spent in the target sector (probe phase) did not differ in the EE and control group animals (*p* = 0.86 for females, and *p* = 0.68 for males). Thus, in spite of the anxiety-like behavior female and male rats showed in the EPM, EE animals were significantly better at finding shelter in the Barnes maze compared to the corresponding control groups, even after a 48 h interval in training.

### 3.2. The Expression Levels of Neurogenesis Markers, Syn1 and IL-10 Increased Only in EE Females

**Figure 3:**
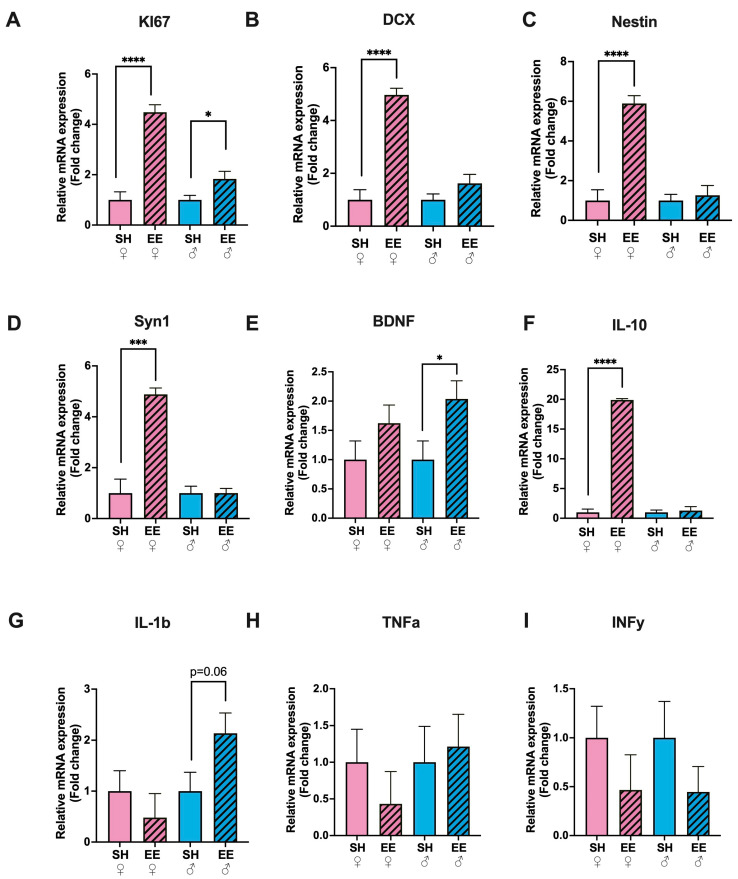
Fold changes in the expression levels of cytokines (IL-1b (**G**), TNFa (**H**), INFy (**I**), IL-10 (**F**)), markers of neurogenesis (KI67 (**A**), DCX (**B**), Nestin(**C**)), and neural plasticity (Syn1 (**D**), BDNF (**E**)) in the hippocampi of EE and SH female (pink) and male (blue) rats. EE: enriched environment, SH: standard housing. ♂—males, ♀—females. A striped pattern of the bars refers to EE, solid fill—to SH conditions. IL-1binterleukin 1b, TNFa—tumor necrosis factor a, INFy—interferon-y, IL-10—interleukin-10, KI67—cell proliferation marker, DCX—doublecortin, Nestin—a type VI intermediate filament protein, Syn1—synapsin 1, BDNF—Brain-derived neurotrophic factor. The values are represented as mean ± SEM, * *p* ≤ 0.05, *** *p* ≤ 0.001, **** *p* ≤ 0.0001 (*t*-test).

In EE rats, the expression level of cell proliferation marker KI67 in the hippocampus increased substantially in females (*p* ≤ 0.0001) and to some extent in males (*p* = 0.03) compared to the corresponding SH groups (Figure 3A), while the expression level of DCX, the marker of immature neurons (Figure 3B), Nestin, a neural stem or progenitor cell marker (Figure 3C), and Syn1, a marker of functional synapses (Figure 3E), increased only in EE females (*p* ≤ 0.0001 for DCX and *p* = 0.0002 for Syn1 in females and *p* = 0.15, *p* = 0.65, *p* = 0.99 for males, respectively). The expression level of brain-derived neurotrophic factor (BDNF) was significantly higher only in EE males compared to SH males (*p* = 0.04), while in EE females it did not change compared to the corresponding control group (*p* = 0.20) (Figure 3D). The processes of adult neurogenesis and synaptic plasticity are influenced by circulating as well as locally produced cytokines; therefore we measured their expression levels in the hippocampi of EE and SH rats (Figure 3F–I). The levels of pro-inflammatory cytokines did not change significantly in EE animals: TNFa (EE vs. SH females: *p* = 0.39; EE vs. SH males: *p* = 0.75), IL-1b (EE vs. SH females: *p* = 0.41; EE vs. SH males: *p* = 0.06) and INFy (EE vs. SH females: *p* = 0.29; EE vs. SH males: *p* = 0.27), while the expression level of anti-inflammatory cytokine IL-10 increased substantially in EE females compared to the corresponding control group (*p* ≤ 0.0001), and in EE males it did not change significantly (*p* = 0.72).

### 3.3. Expression Levels of IL-10Ra, JAK, STAT3 and STAT5a Increased Only in EE Females

**Figure 4:**
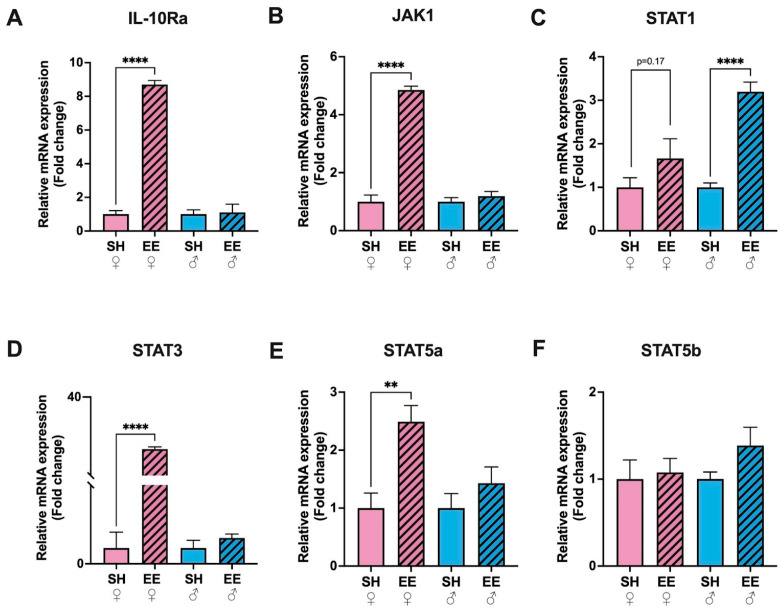
Fold changes in the expression levels of IL-10Ra (**A**) and components of the JAK-STAT signaling pathway: JAK1 (**B**), STAT1 (**C**), STAT3 (**D**), STAT5a (**E**), STAT5b (**F**) in the hippocampi of EE and SH female (pink) and male (blue) rats. EE: enriched environment, SH: standard housing. ♂—males, ♀—females. A striped pattern of the bars refers to EE, solid fill—to SH conditions. The values are represented as mean ± SEM, ** *p* ≤ 0.01,**** *p* ≤ 0.0001 (*t*-test).

The expression level of IL-10Ra increased substantially only in the hippocampi of female EE rats compared to the corresponding control group (*p* ≤ 0.0001; Figure 4), while in males it did not change significantly (*p* = 0.85). The expression levels of the components of the JAK-STAT signaling pathway changed differently in female and male rats: in EE females there was an increased expression on JAK1 (*p* ≤ 0.0001), STAT3 (*p* ≤ 0.0001) and STAT5a (*p* = 0.003), while in males, STAT1 (*p* ≤ 0.0001). JAK1 did not change in EE male rats compared to the corresponding control group (*p* = 0.39), as was the case for STAT3 (*p* = 0.27) and STAT5a (*p* = 0.27). STAT1 did not change in EE females compared to the corresponding control group (*p* = 0.17). The expression of STAT5b stayed the same in both females (*p* = 0.79) and males (*p* = 0.11).

### 3.4. Hippocampal microRNA Expression Profiles Change Differently in Females and Males after EE

**Figure 5:**
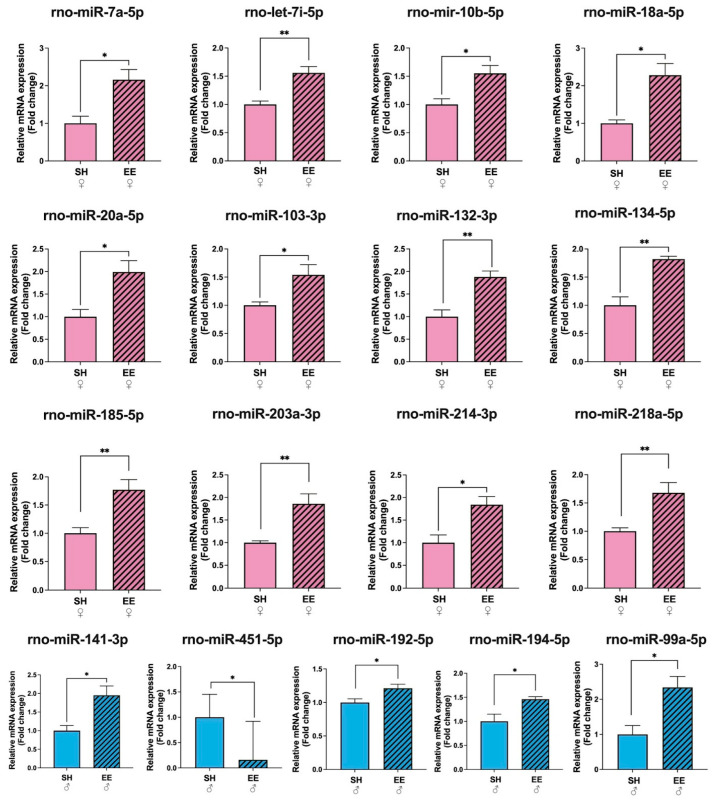
Fold changes in the expression levels of microRNAs in the hippocampi of female and male rats after EE, SNORD95. as a reference gene. EE: enriched environment, SH: standard housing. ♂—males, ♀—females. A striped pattern of the bars refers to EE, solid fill—to SH conditions. Values are represented as mean ± SEM, *n* = 3, * *p* ≤ 0.05, ** *p* ≤ 0.01 (*t*-test).

From 84 miRNAs tested, in the hippocampi of EE female rats we detected changes in the expression levels of 12 miRNAs compared to the corresponding control group, while in EE males, we detected changes in 5 miRNAs when using SNORD95 as a reference gene (Figure 5; Supplementary Table S2). EE females had an increased expression of rno-miR-7a-5p (*p* = 0.02), rno-let-7i-5p (*p* = 0.006), rno-miR-10b-5p (*p* = 0.02), rno-miR-18a-5p (*p* = 0.02), rno-miR-20a-5p (*p* = 0.02), rno-miR-103-3p (*p* = 0.02), rno-miR-132-3p (*p* = 0.009), rno-miR-134-5p (*p* = 0.005), rno-miR-185-5p (*p* = 0.01), rno-miR-203a-3p (*p* = 0.01), rno-miR-214-3p (*p* = 0.02), and rno-miR-218a-5p (*p* = 0.01) in comparison to SH females. In EE males compared to SH males, the expression of four miRNAs increased: rno-miR-99a-5p (*p* = 0.03), rno-miR-141-3p (*p* = 0.02), rno-miR-192-5p (*p* = 0.02), rno-miR-194-5p (*p* = 0.02), and the expression of rno-miR-451-5p decreased (*p* = 0.04) compared to the corresponding control group.

### 3.5. The Number of KI67+ Cells Decreases, While the Number of DCX+ Cells Increases in the Dentate Gyrus of EE Females, with No Such Changes in EE Males

**Figure 6:**
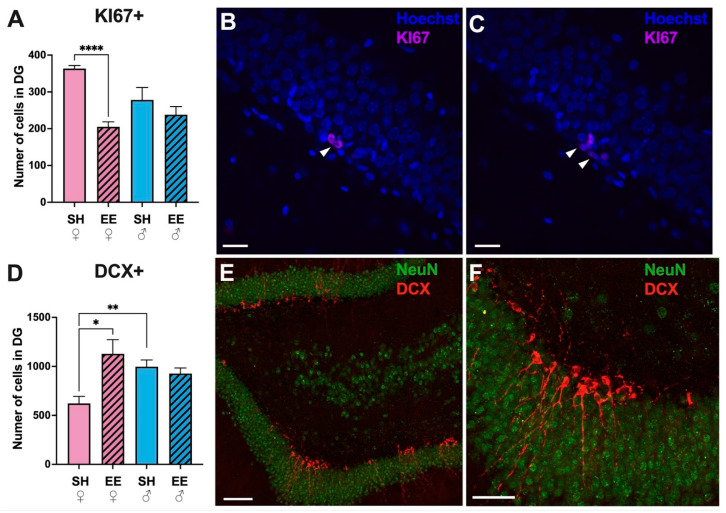
The number of KI67+ (**A**–**C**) and DCX+ (**D**,**E**) cells in the dentate gyrus of the hippocampi of EE and SH female (pink) and male (blue) rats. (**B**,**C**) Representative confocal images of the dentate gyrus of EE female rats stained for the nuclear markers Hoechst (blue) and KI67 (pink). The arrowheads point to stained KI67+ cells. (**E**,**F**) Representative confocal images of the dentate gyrus of EE female rats stained for markers of mature neurons NeuN (green) and markers of immature neurons DCX (red). EE: enriched environment, SH: standard housing. ♂—males, ♀—females. A striped pattern of the bars refers to EE, solid fill—to SH conditions. Values are represented as the mean ± SEM, * *p* ≤ 0.05, ** *p* ≤ 0.01, **** *p* ≤ 0.0001 (*t*-test). Scale bars: 20 μm (**B**,**C**), 100 μm (**E**), 50 μm (**F**).

The immunohistochemical identification of proliferating cells in the dentate gyrus of the hippocampus (Figure 6) revealed that EE significantly decreased the number of KI67+ cells in EE females (*p* = 0.001), while in males no such changes were found (*p* = 0.58). There were also no significant differences in the number of cells in SH females and males (*p* = 0.06). KI67+ cells had a typical bean-shaped morphology, and were located in groups in the subgranular layer of the dentate gyrus (Figure 6B,C). The number of immature DCX+ neurons was higher in EE females than SH females (*p* = 0.01), with no differences between EE and SH males (*p* = 0.45). Notably, SH females had significantly less immature neurons than SH males (*p* = 0.005). DCX+ cells had a typical morphology of immature neurons, with their dendrites passing through the layer of the granular cells of the dentate gyrus, and were located in groups in the subgranular layer (Figure 6E,F).

## 4. Discussion

The enrichment of the laboratory environment in animal facilities supports brain development in the same manner as the natural environment does [16]. An enriched environment is believed to be closer to the natural habitat of rodents, and thus, it is considered to have beneficial effects on learning, memory, and many other brain functions, but the exact molecular mechanisms of these effects are still not fully understood. We have shown that male and female Wistar rats housed in EE conditions for 2 months performed better than SH animals in the Barnes maze, even after a 48 h interval in training, meaning that EE enhances spatial memory. This finding is consistent with previous studies [25,26]. Spatial memory is hippocampus-dependent: both the dorsal and the ventral hippocampus are critical for spatial memory retention [27], while direct projections from the CA1/subiculum of the ventral hippocampus to the medial prefrontal cortex are essential for the encoding, but not for the maintenance or recall of spatial memory [28]. Moreover, the abolishment of adult hippocampal neurogenesis results in deficits in long-term memory in the Morris water maze and the T-maze [29,30,31], meaning that hippocampal neurogenesis is essential for spatial working memory. Experiments on chickadees show that hippocampal neurogenesis aids in pattern separation and the differentiation of similar memories at the time of encoding [32]. Training rats in a Morris water maze supports adult hippocampal neurogenesis, but in a cell stage dependent manner: the enhancement of neurogenesis was detected only when BrdU was injected 6 days before the start of spatial training in the Morris maze [33], i.e., the training had an effect only on neural precursors that were born in a defined time window. Housing rats in EE conditions may be interpreted as a kind of spatial training (EE cages include additional space, ladders, and wheels for voluntary running), but this “training” is long-lasting and excludes the pressure to find shelter in a short time under bright lights, as in a Barnes maze or on platform to keep away from water, as in a Morris maze. In our study, EE males showed better spatial memory retention than females: they were finding shelter faster on two, three, four, and six trials, as well as immediately after the 48 h interval in training (seven and eight trials), while EE females were significantly faster than the corresponding control group only on trials two and six, and after the 48 h break they were not able to recall the position of the shelter on trial seven, but their memory was restored on the eighth trial. Overall, both EE males and females demonstrated less variation in the time they needed to find a shelter than the corresponding SH groups. Previous studies have shown that male rats [34], in addition to humans [35], outperform females in spatial memory abilities, and this becomes more evident as the task difficulty increases. This may be explained by sexual differences in hippocampal neurophysiology as well as connectivity. For example, male rats show larger LTP at the perforant pathway-dentate gyrus synapses than females [34]. Interestingly, male rats perform better on the object location memory task during the night, while females perform better on the task during the day [36], meaning that spatial memory processing also depends on circadian rhythms. According to our protocol for training in a Barnes maze, all animals had two trials per day, i.e., odd numbers of trials (one, three, five, and seven) were performed early in the mornings, while even numbered trials (two, four, six, and eight) were conducted later in the day; and EE females were indeed faster than SH females on trials two, six, and eight, while EE males showed significantly better results on trials two, three, four, five, seven, and eight. It is therefore possible that hippocampal plasticity has sex-specific differences in many aspects, including spatial memory.

According to our results, in the hippocampi of EE female and male rats, the expression level of KI67 was higher than in the corresponding SH groups, indicating an increase in cell proliferation as a result of being housed in EE conditions; however, the expression level of DCX and Nestin only increased in EE females, suggesting that at the time of PCR analysis, adult hippocampal neurogenesis increased only in female EE rats. It has been shown that regular physical activity stimulates the proliferation of neural precursors in the adult hippocampus [37,38,39]. However, the increased mRNA level of KI67 may also indicate the proliferation of glial or endothelial cells, i.e., ongoing angiogenesis. It was shown earlier that running increases VEGF-signaling and blood supply to the dentate gyrus [40]. Immunohistochemical data confirmed our conclusions: the number of KI67+ cells in the subgranular layer of the dentate gyrus decreased in EE females, with no significant changes in EE males, and the number of DCX+ cells significantly increased only in EE females. The decrease in KI67+ cells in EE females may be explained by their differentiation into DCX+ immature neurons. The data on female rats are supported by other studies, indicating that housing in an enriched environment stimulates adult hippocampal neurogenesis [41,42], while our results on male rats are rather unexpected and may be explained either by the difference in neural plasticity dynamics in males and females (for example, new neurons had been created earlier; this is further supported by our finding that SH males had significantly more DCX+ cells than SH females), or by the effect of stress due to strict social hierarchy in males. In a socially enriched environment, rats and mice develop a strict social hierarchy, with dominant and subordinate males. The agonistic behavior of the dominant male promotes the social hierarchy and avoidance of fights; however, if the subordinate male does not respond with appropriate submissive behavior, the dominant one resorts to violence, so submissive or socially low-ranking animals may suffer from stress, show immunodeficiency, and be more susceptible to viral infections and the formation of tumors (Brown and Grunberg, 1995). Unfortunately, we were unable to monitor the social hierarchy in males, since housing in EE conditions lasted for 2 months, and the three-tiered cages we used for housing did not allow us to observe all male-to-male contacts. Furthermore, from our observations in mice, it was evident that the separation of a dominant aggressive males usually did not solve the problem, because another male will likely become dominant. We have shown previously that stress decreases the level of adult hippocampal neurogenesis in C57BL/6 mice [43]. When animals experience stress, one might expect that the levels of inflammatory markers increase in the blood and in the brain [44], which can affect the level of hippocampal neurogenesis [45]. However, in measuring the expression levels of cytokines in the hippocampal tissue, we have found no significant differences in the expression of proinflammatory TNFa and INFy, with only a slight tendency to increase for IL-1b in EE males, and thus there were no signs of ongoing neuroinflammation. At the same time, since male EE rats did not show significant signs of anxiety in the EPM (it was rather females who we considered to be anxious), we cannot conclude that they were all stressed. Nevertheless, since male EE rats showed significantly better results on the Barnes maze than SH males, we can suggest that either the dynamics of neurogenesis is different in males compared to females in EE conditions, or their spatial memory is less dependent as the result of hippocampal neurogenesis. Thus, the expression level of hippocampal BDNF was significantly higher in EE males compared to SH males, and both EE and SH female rats. It is well-known that BDNF is involved in the regulation of activity-dependent forms of synaptic plasticity such as LTP [46].

Interestingly, the expression level of anti-inflammatory cytokine IL-10 was significantly higher in EE females compared to SH females and both EE and SH males, and it was the only cytokine which showed difference in the expression after housing in EE conditions. IL-10 is known as a core inhibitor of IKKβ/NF-κB signaling and ER stress [47]; its downstream signaling via STAT3 results in the downregulation of MHCII and cytokine gene expression, reducing inflammation [48]. It was shown earlier that IL-10 regulates progenitor differentiation [49] and adult neurogenesis by modulating ERK and STAT3 activity in the adult subventricular zone; IL-10 acts on Nestin+ neural progenitors via IL-10Ra, promoting cell proliferation [50]. We found an increased expression level of Nestin in the hippocampi of EE females compared to the corresponding control group, and further analysis showed that the expression levels of IL-10Ra, JAK1, STAT3, and STAT5a were significantly higher in EE females, while the expression of STAT1 was higher in EE males. However, we cannot yet make a conclusion about the role of IL-10 in the adult hippocampal neurogenesis in EE conditions; we can only state that EE enhances IL-10 mRNA expression in the rat hippocampus in a sex-dependent manner. It is possible that housing in EE conditions has an anti-inflammatory effect on the brain. For example, an enriched environment restores metabolic homeostasis by reducing obesity-induced inflammation [51], and inhibits neuroinflammation and oxidative stress after a stroke [52]. Thus, IL-10 might have an anti-inflammatory effect on hippocampal glial cells, which is favorable for neurogenesis progression. Since all animals in our study underwent transcardial perfusion with saline, and all peripheral blood was removed from the brain, we consider the source of IL-10 mRNA to be glial cells, i.e., microglia. However, in order to make a conclusion about the role and the source of IL-10 in the hippocampus, this cytokine needs to be measured on a protein level.

An analysis of the miRNA profiles also showed striking differences in male and female rats (below we discuss the results from using SNOR95 as a reference gene, if not mentioned otherwise). In EE females we found changes in the expression level of 12 miRNAs (rno-miR-7a-5p, rno-let-7i-5p, rno-miR-10b-5p, rno-miR-18a-5p, rno-miR-20a-5p, rno-miR-103-3p, rno-miR-132-3p, rno-miR-134-5p, rno-miR-185-5p, rno-miR-203a-3p, rno-miR-214-3p, and rno-miR-218a-5p increased), while in EE males, only five miRNAs showed changes (rno-miR-99a-5p, rno-miR-141-3p, rno-miR-192-5p and rno-miR-194-5p increased, while rno-miR-451-5p decreased); some of their known functions are summarized in Table 2.

We found an increased expression of miRNAs, regulating neuronal differentiation: miR-let-7i-5p [53,54], miR-7a-5p [55], miR-20a-5p [64], miR-132-3p [66], miR-185-5p [75], miR-218a-5p [79] and neuronal morphogenesis: miR-103-3p [65], miR-132-3p [67,68,69], miR-134-5p [72,73], miR-185-5p [76], miR-214-3p [78], and miR-218a-5p [80] in the hippocampi of female rats. For example, miR-132 is known to be enriched in the brain compared to other organs in humans and mice [89], and its functions range from stimulation of dopaminergic neuron differentiation [66] and the promotion of axonal branching [67] to the enhancement of spatial memory and cognitive capacity [71]. It is seemingly one of the miRNAs that regulates experience-dependent neuronal plasticity [70]. Thus, an increase in the expression of the above-mentioned microRNAs indicates active neurogenesis and experience-dependent neuronal plasticity in the hippocampus of EE females compared to the corresponding SH female rats. Other miRNAs enriched in EE females included those involved in oligodendrocyte differentiation: miR-18a-5p [63] and NF-κβ signaling pathway regulation, and microglia polarization: miR-7a-5p [56,57], miR-10b-5p [60], and miR-203a-3p [77]. White matter plasticity is an important mechanism that is essential in supporting adult brain functioning [90], while the regulation of NF-κβ signaling and corresponding inflammation via miRNAs and/or IL-10 protects brain cells from oxidative stress [52]. Interestingly, arranging our miRNA data according to another reference gene (SNOR72) revealed the upregulation of one miRNA in both EE males as well as EE females, that being miR-146a-5p (Supplementary Table S2), which is known to be upregulated in monocytes producing IL-10 [91]. It was recently shown that microglia secrete miR-146a-5p-containing exosomes to regulate neurogenesis in depression [92].

**Figure 7:**
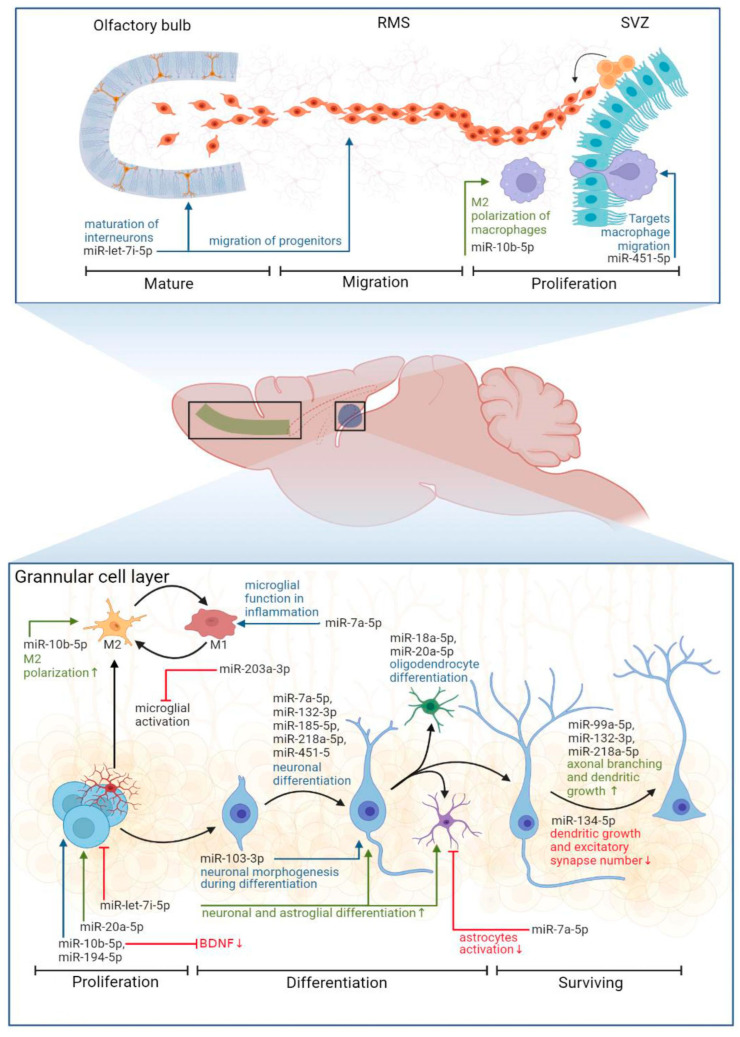
Proposed functions of identified miRNAs (SNOR95 as a reference gene) in the processes of adult glio- and neurogenesis [53,54,55,56,57,58,59,60,61,62,63,64,65,66,67,68,69,70,71,72,73,74,75,76,77,78,79,80,81,82,83,84,85,86,87,88]. RMS: rostral migratory stream, SVZ: subventricular zone, M1 and M2: pro- and anti-inflammatory phenotypes of microglial cells, BDNF: brain-derived neurotrophic factor. BioRender was used to build this image.

We identified miRNAs in the hippocampus of EE males that regulate neuronal proliferation and apoptosis: these being miR-194-5p [85] and miR-141-3p [83]; neuronal differentiation in miR-451-5p [87]; neurite growth in miR-99a-5p [81]; the inhibition of LPS-induced astrocytes activation in miR-194-5p [86]; and maintaining cognitive function in the mice model of depression: miR-192-5p [84]. From the array of identified miRNA in males we cannot conclude that adult hippocampal neurogenesis is stimulated by EE in male rats, since miRNA functions vary. For example, the upregulation of miR-194-5p promotes cell viability and inhibits the apoptosis of hippocampal neurons [85], while the upregulation of miR-141-3p is shown to inhibit the neurogenesis of rat neural stem cells [83]. The upregulation of miR-99a-5p suppressed the vitality, proliferation, and migration of human oral carcinoma cells [82]. An increase in miR-451-5p expression is associated with the acceleration of neuronal differentiation, while a decrease in expression (which is the case in EE males according to our data) is associated with the stimulation of proliferation versus neuronal differentiation [87]. Arranging the data according to other reference genes (SNOR61 and SNOR68) revealed the significant downregulation of miR-451-5p expression as well (Supplementary Table S2). Thus, microRNA profiling of the hippocampus revealed significant changes in the expression levels of microRNAs associated with glio- and neurogenesis, as well as other forms of neuronal plasticity (Figure 7).

Taken altogether, our results point to sex-specific differences in the adult hippocampal plasticity, IL-10 expression, and miRNA profiles induced by an enriched environment.

## 5. Conclusions

The adult brain is responsive to environmental enrichment, and we have shown that housing male and female Wistar rats in EE conditions for 2 months enhances their spatial memory, with males performing better than females in a Barnes maze. However, an enriched environment induced sex-specific changes in the hippocampal plasticity in the expression levels of neurogenesis markers KI67, DCX and Nestin, and Syn1 only increased in EE females, while in EE males only proliferation rates (KI67) and BDNF were higher than in the corresponding control. The immunohistochemical data supported the PCR results: the number of DCX+ neurons increased in the dentate gyrus of EE females, with no such changes seen in EE males, i.e., the level of adult hippocampal neurogenesis was increased in females but not in male rats after housing in EE conditions. The expression levels of the proinflammatory cytokines TNFa, INFy and IL-1b did not change significantly in either females or males, while the level of anti-inflammatory IL-10 as well as IL-10Ra, JAK1, STAT3 and STAT5a was upregulated in EE females, with no such changes in EE males. IL-10 might have an anti-inflammatory effect on hippocampal glial cells, which is favorable for neurogenesis progression. From 84 miRNAs tested, in the hippocampi of EE female rats we detected an upregulation in the expression levels of 12 miRNAs related to neuronal differentiation and morphogenesis, oligodendrocyte differentiation, and the regulation of inflammation, while in EE males, four miRNAs were upregulated and involved in the regulation of cell proliferation/differentiation, and one was downregulated, which is associated with the stimulation of proliferation versus neuronal differentiation. Thus, microRNA profiling of the hippocampus revealed significant changes in the expression levels of microRNAs associated with neurogenesis and other forms of neuronal plasticity.

## Figures and Tables

**Table 1:** Primers used in the study.

Gene	Accession No.	Forward (5′-3′)	Reverse (5′-3′)	Amplicon, bp
Ki-67	NM_001271366.1	GCACAGAGCCTTAGCAATAG	GGTGCTTCTACTGGACTTTG	198
DCX	NM_053379.3	CTCAAGCCAGAGAGAACAAG	GCTTTCCATCAAGGGTATAGAG	201
Nestin	NM_001308239.1	AGGAGTGGGAACTGAGGATAAG	TGAGCAACTGGGACCTCTAA	201
IL-10 [23]	NM_012854.2	TAAGGGTTACTTGGGTTGCC	TATCCAGAGGGTCTTCAGC	142
IL-10RA	NM_057193.2	CCCATGAACTTGTCCCTCTG	GAAACCTTATCCCCTGTCACTC	129
JAK1	NM_053466.1	GGACACTGGACAACCGAATAA	TTGTGGCAGAGAGGAGAGATA	248
STAT1	NM_032612.3	TTGAGCCCTACACGAAGAAAG	GGTGGACTTCAGACACAGAAA	249
STAT3	NM_012747.2	GGGCATCAATCCTGTGGTATAA	GGAATGTCAGGGTAGAGGTAGA	613
STAT5A	NM_017064.2	AGGAAGGGAGGCAAGTTTATG	CCGCAGCCCATATTCACTAA	182
STAT5B	NM_022380.2	CAGTTCAGTGTTGGTGGAAATG	CCAGTGAGGCTTGAGATGTT	429
IL-1β	NM_031512.2	GCAATGGTCGGGACATAGTT	GTAAGTGGTTGCCTGTCAGAG	242
TNF-α	NM_012675.3	GAACAGCAACTCCAGAACA	CACGAGCAGGAATGAGAAG	243
IFN-γ	NM_138880.3	ATCTCTTTCTACCTCAGACTCTTTG	TTGCTTTACTGTTGCTGAAGAAG	115
BDNF	NM_001270638.1	GAGACAAGAACACAGGAGGAAA	CCCAAGAGGTAAAGTGTAGAAGG	106
Syn1	NM_019133.2	CCTCTTCAAATGCCACCTACTA	GGTTTCTGGAGGAAGGAACTTA	133
GAPDH	NM_017008.4	GCTGTGGGCAAGGTCATCC	CTTCACCACCTTCTTGATGTC	144

**Table 2:** Functions of identified miRNAs.

miRNA	Function	Fold Change/*p*-Value Based on Our Results (SNOR95)
**Females**		
miR-let-7i-5p	Reduces proliferation and promotes both neuronal and astroglial differentiation [53].	1.56/0.006
	Crucial for the functional radial migration and maturation of olfactory bulb interneurons [54].	
miR-7a-5p	Inhibits Pax6 and promotes differentiation of dopaminergic neurons [55].	2.16/0.02
	Regulates the function of microglia in inflammation [56].	
	Promotes E2F-mediated cell proliferation and NFκB activation in vitro [57].	
miR-10b-5p	Mediates cell communication of fibroblasts and facilitates cell proliferation [58].	1.55/0.02
	Biomarker in Huntingtons disease [59].	
	Enhances M2 polarization of macrophages [60].	
	Overexpression reduces BDNF expression and elevated apoptosis rate in vitro [61].	
	Upregulation leads to decreased BDNF levels in mice [62].	
miR-18a-5p	Regulates oligodendrocyte differentiation [63].	2.28/0.02
miR-20a-5p	Inhibits CCND1 and promotes neuronal differentiation [64]. Regulates oligodendrocyte differentiation [63].	1.99/0.02
miR-103-3p	Regulates morphogenesis of new neurons during their differentiation [65].	1.54/0.02
miR-132-3p	Inhibits Nurr1 and promotes differentiation of dopaminergic neurons [66]. Promotes axonal branching by inhibiting the translation of p250GAP [67].	1.88/0.009
	Regulates the dendritic growth and branching of young hippocampal neurons in vitro and in vivo [67,68,69].	
	Involved in experience-dependent visual cortex plasticity [70].	
	Moderate overexpression of miR-132 enhanced spatial memory and cognitive capacity in Barnes maze/novel object recognition task, while supra-physiological level of miR-132 impaired cognition [71].	
	Expression changes in the hippocampus after training are essential for LTP [69].	
miR-134-5p	Inhibits dendritic development [72].	1.82/0.005
	Overexpression in the rat mPFC decreases dendritic spine density and synapse number [73].	
	Activity-induced hippocampal dendritic growth and excitatory synapse number [74].	
miR-185-5p	Regulates neuronal differentiation of neural progenitors [75] and dendritic spine morphology [76].	1.77/0.01
miR-203a-3p	Inhibits NF-κβ signaling pathway activation and microglia activation [77].	1.86/0.01
miR-214-3p	Decreases amplitude of mEPSC, and number of dendritic spines in hippocampal neurons [78].	1.84/0.02
miR-218a-5p	Promotes neuronal differentiation [79].	1.68/0.01
	Overexpression increases density of dendritic spines the mPFC [80].	
**Males**		
miR-99a-5p	Regulates neurite growth in mouse model of spinal injury [81].	2.34/0.03
	Regulates proliferation, migration and invasion of human carcinoma cells [82].	
miR-141-3p	Inhibits neurogenesis [83].	1.95/0.02
miR-192-5p	Enhances cognitive function in mice model of depression [84].	1.21/0.02
miR-194-5p	Regulates proliferation and apoptosis of hippocampal neurons in children with temporal lobe epilepsy [85].	1.46/0.02
	Inhibits LPS-induced astrocytes activation [86].	
miR-451-5p	Overexpression induces accelerated neuronal differentiation [87].	0.16/0.04
	Targets macrophage migration [88].	

## Data Availability

The data presented in this study are available upon request from the corresponding author.

## Supplementary Materials

The following are available online at https://www.mdpi.com/article/10.3390/biomedicines11051341/s1, Table S1: Number of animals used in each experimental group. Table S2: changes in mRNA expression in females, reference gene—SNORD61; changes in mRNA expression in females, reference gene—SNORD68; changes in mRNA expression in females, reference gene—SNORD72; changes in mRNA expression in females, reference gene—SNORD95; changes in mRNA expression in females, reference gene—SNORD96A; changes in mRNA expression in females, reference gene—RNU6-6P; changes in mRNA expression in males, reference gene—SNORD61; changes in mRNA expression in males, reference gene—SNORD68; changes in mRNA expression in males, reference gene—SNORD72; changes in mRNA expression in males, reference gene—SNORD95; changes in mRNA expression in males, reference gene—SNORD96A; changes in mRNA expression in males, reference gene—RNU6-6P. The table shows the tested miRNAs and the fold change.
