# Supplementary material for: Enriched Environment Induces Sex-Specific Changes in the Adult Neurogenesis, Cytokine and miRNA Expression in Rat Hippocampus
Source: Biomedicines. 2023 May 2;11(5):1341. doi: 10.3390/biomedicines11051341 (PMC10215805; doi:10.3390/biomedicines11051341)
Supplement: Supplementary file 1 [file biomedicines-11-01341-s001.zip › biomedicines-2262074-supplementary.pdf]

Table S1. Number of animals used in each experimental group.

Experiment	Group	Male/Female	Number of Animals
Open field	Before Control (age - P30)	Male	8
		Female	8
	Before EE (age - P30)	Male	7
		Female	6
	Control (age - P90)	Male	24
		Female	23
	EE (age - P90)	Male	27
		Female	28
Elevated plus maze	Control (age - P90)	Male	24
		Female	23
	EE (age - P90)	Male	23
		Female	23
Barnes maze	Control (age - P90)	Male	14
		Female	13
	EE (age - P90)	Male	17
		Female	16
PCR: KI67, DCX, Nestin, BDNF	Control (age - P90)	Male	6
		Female	7
	EE (age - P90)	Male	6
		Female	5
PCR: IL-10, IL-10RA	Control (age - P90)	Male	6
		Female	6
	EE (age - P90)	Male	6
		Female	5
PCR: JAK1	Control (age - P90)	Male	6
		Female	6
	EE (age - P90)	Male	6
		Female	5

PCR: STAT1, STAT5A, STAT5B	Control (age - P90)	Male	6
		Female	7
	EE (age - P90)	Male	6
		Female	5
PCR: STAT3	Control (age - P90)	Male	6
		Female	5
	EE (age - P90)	Male	6
		Female	5
PCR: microRNA	Control (age - P90)	Male	3
		Female	3
	EE (age - P90)	Male	3
		Female	3
Immunohistochemistry	Control (age - P90)	Male	7
		Female	5
	EE (age - P90)	Male	7
		Female	5

Table S2. Changes in mRNA expression in females, reference gene—SNORD61; changes in mRNA expres-sion in females, reference gene—SNORD68; changes in mRNA expression in fe-males, reference gene—SNORD72; changes in mRNA expression in females, reference gene—SNORD95; changes in mRNA expression in females, reference gene—SNORD96A; changes in mRNA expression in females, reference gene—RNU6-6P; changes in mRNA expression in males, reference gene—SNORD61; changes in mRNA expression in males, reference gene—SNORD68; changes in mRNA expression in males, reference gene—SNORD72; changes in mRNA expression in males, reference gene—SNORD95; changes in mRNA expression in males, reference gene—SNORD96A; changes in mRNA expression in males, reference gene—RNU6-6P. The table shows the tested miRNAs and the fold change.

		**Female**	**Male**	**Female**	**Male**	**Female**	**Male**	**Female**	**Male**	**Female**	**Male**	**Female**	**Male**
		**rno-let-7a-5p**		**rno-miR-7a-5p**		**rno-let-7i-5p**		**rno-miR-10b-5p**		**rno-miR-18a-5p**		**rno-miR-20a-5p**	
**Reference Gene**	SNORD61	-	-	-	-	-	-	-	-	-	-	-	-
	SNORD68	0.50/ 0.004	-	-	-	-	-	-	-	-	-	-	-
	SNORD72	-	-	-	-	-	-	-	-	1.74/ 0.03	-	-	-
	SNORD95	-	-	2.16/ 0.02	-	1.56/ 0.006	-	1.55/ 0.02	-	2.28/ 0.02	-	1.99/ 0.02	-
	SNORD96A	-	-	-	-	-	-	-	-	-	-	-	-
	RNU6-6P	-	-	-	-	-	-	-	-	-	-	-	-
		Female	Male	Female	Male	Female	Male	Female	Male	Female	Male	Female	Male
		rno-miR-26a-5p		rno-miR-99a-5p		rno-miR-103-3p		rno-miR-125a-5p		rno-miR-132-3p		rno-miR-134-5p	

**Reference Gene**	SNORD61	-	-	-	-	-	-	-	-	-	-	-	-
	SNORD68	0.51/ 0.04	-	-	-	-	-	0.63/ 0.001	-	-	-	-	-
	SNORD72	-	-	-	-	-	-	-	-	-	-	-	-
	SNORD95	-	-	-	2.34/ 0.03	1.54/ 0.02	-	-	-	1.88/ 0.009	-	1.82/ 0.005	-
	SNORD96A	-	-	-	-	-	-	-	-	-	-	-	-
	RNU6-6P	-	-	-	-	-	-	-	-	-	-	-	-

		Female	Male	Female	Male	Female	Male	Female	Male	Female	Male	Female	Male
		rno-miR-141-3p		rno-miR-144-3p		rno-miR-146a-5p		rno-miR-185-5p		rno-miR-192-5p		rno-miR-194-5p	
**Reference Gene**	SNORD61	-	1.36/ 0.03	-	0.21/ 0.04	-	-	-	-	-	-	-	-
	SNORD68	-	-	-	-	-	-	-	-	-	-	-	-
	SNORD72	-	-	-	-	1.29/ 0.01	1.56/ 0.01	-	-	-	-	-	-
	SNORD95	-	1.95/ 0.02	-	-	-	-	1.77/ 0.01	-	-	1.21/ 0.02	-	1.46/ 0.02
	SNORD96A	-	-	-	-	-	-	-	-	-	-	-	-
	RNU6-6P	-	-	-	-	-	-	-	-	-	-	-	-

		Female	Male	Female	Male	Female	Male	Female	Male	Female	Male
		rno-miR-203a-3p		rno-miR-205		rno-miR-214-3p		rno-miR-218a-5p		rno-miR-451-5p	
**Reference Gene**	SNORD61	-	-	0.5/ 0.040	-	-	-	-	-	0.34/ 0.02	0.11/ 0.02
	SNORD68	-	-	-	-	-	-	-	-	0.31/ 0.008	2.77/ 0.04
	SNORD72	-	-	-	-	-	-	-	-	-	0.14/ 0.02
	SNORD95	1.86/ 0.01	-	-	-	1.84/ 0.02	-	1.68/ 0.01	-	-	0.16/ 0.04
	SNORD96A	-	-	-	-	-	-	-	-	-	0.15/ 0.03
	RNU6-6P	-	-	0.63/ 0.003	-	-	-	-	-	-	-
